# Time-Dependent Transition in the Re-Tempering Behavior of Cement Mortar

**DOI:** 10.3390/ma19153235

**Published:** 2026-07-30

**Authors:** Jun-Cheol Lee

**Affiliations:** Department of Architecture, Seowon University, Cheongju 28674, Republic of Korea; leejc@seowon.ac.kr; Tel.: +82-43-299-8788

**Keywords:** cement mortar, re-tempering, time-dependent transition, workability recovery, compressive strength

## Abstract

Construction delays frequently reduce the workability of cement mortar, and re-tempering by adding extra water is commonly practiced to restore its fluidity. Although re-tempering improves fresh-state workability, it may simultaneously impair the hardened properties of mortar. This study investigated the combined effects of waiting time (0, 15, 30, and 45 min) and additional water content (0, 2, and 4% of the initial mixing water) on the fresh and hardened properties of cement mortar under controlled laboratory conditions. Mortar flow, compressive strength, and 28-day water absorption were evaluated to characterize the re-tempering behavior. Re-tempering effectively restored workability; however, its recovery efficiency decreased with increasing waiting time. In contrast, compressive strength decreased, and water absorption increased progressively as both waiting time and additional water content increased. To quantitatively evaluate the balance between workability recovery and strength deterioration, a dimensionless Workability–Strength Trade-off Ratio (WSTR) was introduced as a comparative indicator based on normalized flow recovery and normalized 28-day strength loss. WSTR consistently decreased with increasing waiting time, with a marked reduction after approximately 30 min, indicating that the engineering benefit of re-tempering diminished as hydration progressed. These findings demonstrate that the dominant effect of re-tempering gradually shifts from workability recovery to deterioration of hardened properties, providing a quantitative framework for evaluating re-tempering under construction delay conditions. The findings should be interpreted within the investigated experimental conditions, and further studies involving different mortar mixtures and material systems are required to validate the proposed interpretation.

## 1. Introduction

Cement-based mortars are widely used in architectural finishing applications, such as plastering, masonry, and tile installation. Their performance depends not only on adequate workability in the fresh state but also on sufficient mechanical properties after hardening. However, because mortar is generally mixed and consumed immediately on construction sites, it is highly susceptible to workability loss caused by construction delays [1,2,3]. Once water is added, cement hydration begins immediately, and the formation of early hydration products together with particle flocculation progressively reduces the fluidity of the mixture [4,5]. Such workability loss not only decreases construction efficiency and finishing quality but also results in material waste due to the disposal of unused mortar, leading to unnecessary economic losses.

To restore the reduced workability, the addition of extra mixing water, commonly referred to as re-tempering, is frequently practiced on construction sites. Re-tempering temporarily improves workability by increasing the fluidity of the mortar, thereby facilitating placement and finishing operations. However, the additional water also increases the effective water-to-cement ratio and disturbs the developing hydration structure, which may adversely affect the mechanical strength and durability of the hardened mortar [6]. Consequently, re-tempering inherently involves a trade-off between the recovery of fresh-state workability and the deterioration of hardened-state performance. Appropriate application therefore requires consideration of both beneficial and detrimental effects.

Previous studies on re-tempering have primarily focused on ready-mixed concrete, where additional water has been used to compensate for slump loss during transportation [7,8,9,10,11,12]. Most investigations have evaluated the influence of either the waiting time before re-tempering or the amount of additional water as independent variables, whereas the combined effects of these two parameters have received comparatively little attention [13]. Furthermore, although mortar generally has a shorter workable period than concrete and is more directly affected by construction delays, systematic investigations into the re-tempering behavior of cement mortar remain limited.

More importantly, previous studies have generally interpreted the effects of re-tempering in terms of individual material properties, such as increased workability or reduced compressive strength [14,15]. In practice, however, the dominant effect of re-tempering is expected to evolve with elapsed time. At early ages, the added water effectively restores the lost workability. As hydration progresses, however, the same amount of additional water becomes progressively less effective in recovering workability, while the deterioration of hardened properties caused by increased porosity and disruption of the developing cementitious matrix becomes more pronounced [4,16,17,18]. Accordingly, the dominant influence of re-tempering may gradually shift from workability recovery to deterioration of hardened properties, representing a time-dependent transition. This perspective provides a more comprehensive framework for understanding re-tempering as a time-dependent material response rather than simply the addition of water, allowing the trade-off between fresh and hardened properties to be interpreted in an integrated manner.

In this study, all experiments were conducted under controlled laboratory conditions with constant temperature and relative humidity to isolate the influence of elapsed time. Waiting time before re-tempering (0, 15, 30, and 45 min) and additional water content (0, 2, and 4% of the initial mixing water) were selected as the experimental variables. Mortar flow, compressive strength, and water absorption were systematically evaluated to investigate the combined effects of waiting time and re-tempering water content on both fresh and hardened properties. Furthermore, a dimensionless Workability–Strength Trade-off Ratio (WSTR) was introduced as a comparative indicator to quantitatively assess the balance between workability recovery and strength deterioration under identical experimental conditions. Based on these results, the re-tempering behavior of cement mortar was interpreted as a time-dependent transition from workability recovery to deterioration of hardened properties. The objective of this study is not to identify a specific transition time, but rather to demonstrate the existence of this time-dependent transition and to quantitatively describe it using the proposed Workability–Strength Trade-off Ratio (WSTR).

## 2. Materials and Methods

### 2.1. Materials

Ordinary Portland cement (OPC) conforming to KS L 5201 was used as the binder, and standard sand specified by KS L 5100 was employed as the fine aggregate [19,20]. Mortar mixtures were prepared in accordance with KS L 5105 using a cement-to-sand mass ratio of 1:3 and a fixed water-to-cement ratio (w/c) of 0.50 [21].

The reference mortar was prepared in the laboratory using these constituent materials, and no chemical admixtures or other additives were used in any of the mortar mixtures.

All specimens were produced using the same constituent materials and mix proportions. The only experimental variables were the waiting time before re-tempering and the amount of additional water. The mixture design and experimental variables are summarized in Table 1.

### 2.2. Experimental Design

To isolate the effect of elapsed time on the re-tempering behavior of cement mortar, all experiments were conducted under controlled laboratory conditions at a temperature of 21 ± 1 °C and a relative humidity of 50%.

A two-factor experimental design was adopted using waiting time and additional water content as the independent variables. The waiting time after the completion of initial mixing was set to 0, 15, 30, and 45 min, while the additional water content was fixed at 0, 2, and 4% of the initial mixing water. The waiting times of 15, 30, and 45 min were selected to represent relatively short construction delays that may occur during practical mortar placement and finishing operations. The additional water contents of 2% and 4% were selected to represent relatively small re-tempering water additions that may reasonably be applied in practical mortar operations to restore workability while minimizing changes to the original mixture composition. Accordingly, ten mortar mixtures were prepared, as listed in Table 1.

### 2.3. Re-Tempering Procedure

Immediately after the initial mixing, the mortar was stored under controlled laboratory conditions until the designated waiting time had elapsed. All mixtures were then remixed for 30 s. During the waiting period, the mortar mixtures were stored under controlled laboratory conditions (21 ± 1 °C and 50% relative humidity) without surface covering.

For the re-tempered mixtures, additional water corresponding to 2% or 4% of the initial mixing water was introduced immediately before remixing. The control mixtures (R0) were remixed under identical conditions without adding extra water. Accordingly, the nominal water-to-cement ratio increased from 0.50 to approximately 0.51 and 0.52 for the mixtures containing 2% and 4% additional water, respectively.

Following remixing, the mortar flow test was conducted in accordance with KS L 5111, after which specimens for compressive strength testing were prepared [22]. The specimens were stored at 21 ± 1 °C and 50% relative humidity for 24 h, demolded, and subsequently cured in water at 21 ± 1 °C until testing at 3, 7, and 28 days.

### 2.4. Test Method

The workability of the mortar was evaluated using the flow test specified in KS L 5111. The test was performed immediately after remixing, and the flow diameter (mm) was recorded for each mixture.

Compressive strength was measured at curing ages of 3, 7, and 28 days in accordance with KS L ISO 679 [23]. Three specimens were tested for each mixture at each curing age, and the average value was reported.

Water absorption was determined in accordance with KS F 2476 using specimens cured in water for 28 days [24]. Water absorption was measured using 50 × 50 × 50 mm mortar specimens, and the same specimen type was used for all mortar mixtures. The saturated mass (*W_sat_*) was first measured, after which the specimens were oven-dried at 100 °C for 24 h to obtain the oven-dry mass (*W_dry_*). The water absorption was calculated using Equation (1).

(1) $$Water\;absorption\;(\%)=\frac{W_{sat}-W_{dry}}{W_{dry}}\times 100$$

## 3. Results and Discussion

### 3.1. Effect of Waiting Time and Re-Tempering on Mortar Flow

**Figure 1:**
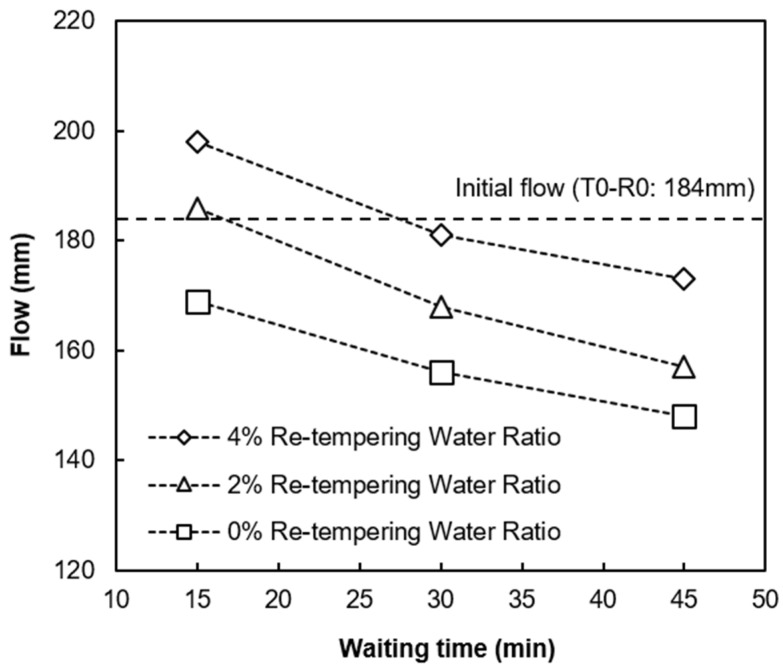
Effect of waiting time and re-tempering water content on mortar flow.

Figure 1 presents the flow of mortar as a function of waiting time and additional water content. For the control mixtures without re-tempering (R0), the flow decreased continuously as the waiting time increased. The initial flow of the reference mixture (T0-R0) was 184 mm, which decreased to 169, 156, and 148 mm after waiting times of 15, 30, and 45 min, respectively. This result indicates the progressive loss of workability caused by the continued hydration and flocculation of cement particles during the waiting period.

The re-tempered mixtures exhibited a clear recovery in workability owing to the addition of extra water. With an additional water content of 2% (T15-R2, T30-R2, and T45-R2), the flow values increased to 186, 168, and 157 mm, respectively, which were consistently higher than those of the corresponding control mixtures. Increasing the additional water content to 4% further improved the flow to 198, 181, and 173 mm for waiting times of 15, 30, and 45 min, respectively, demonstrating that greater amounts of additional water resulted in greater recovery of workability.

Nevertheless, the effectiveness of re-tempering gradually diminished with increasing waiting time. For example, the flow of the mixtures containing 4% additional water decreased from 198 mm at 15 min to 181 mm at 30 min and further to 173 mm at 45 min. A similar tendency was observed for the mixtures containing 2% additional water. Although re-tempering restored the workability lost during the waiting period, the magnitude of recovery became progressively smaller as the hydration process advanced.

In particular, the addition of 2% water almost restored the initial workability at a waiting time of 15 min, yielding a flow of 186 mm comparable to that of the reference mixture (184 mm). However, the recovered flow decreased to 168 and 157 mm after waiting times of 30 and 45 min, respectively. Although the addition of 4% water consistently produced the highest flow values, the same decreasing trend with waiting time remained evident. These results indicate that re-tempering provides only a temporary recovery of workability, and its effectiveness becomes increasingly limited as the elapsed time before re-tempering increases. This behavior is consistent with the progressive hydration and flocculation of cement particles, which continuously reduce the mobility of the fresh mortar and limit the effectiveness of additional mixing water [1,2,3,4,5].

### 3.2. Transition from Workability Recovery to Deterioration of Hardened Properties

**Figure 2:**
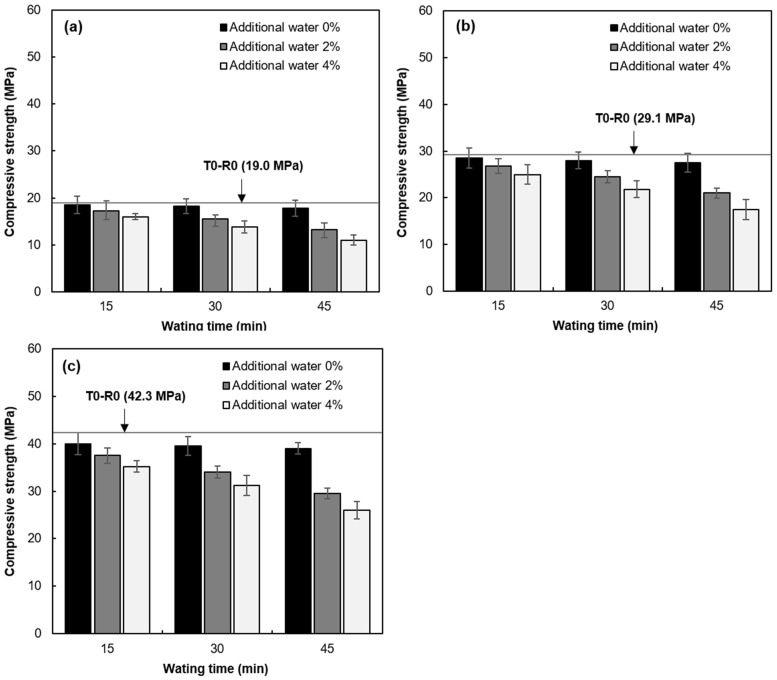
Compressive strength of mortar as affected by waiting time and additional water content: (**a**) 3 days, (**b**) 7 days, and (**c**) 28 days. The horizontal line indicates the compressive strength of the reference specimen (T0-R0). Error bars indicate standard deviations (*n* = 3).

As demonstrated in Section 3.1, re-tempering effectively restored the workability lost during the waiting period. However, comparison of the hardened properties under the same experimental conditions revealed that this improvement in workability was accompanied by a deterioration in compressive strength. Figure 2 presents the compressive strengths of the mortar mixtures measured after 3, 7, and 28 days of curing.

For the control mixtures (R0), compressive strength gradually decreased with increasing waiting time at all curing ages. The 3-day compressive strength decreased from 19.0 MPa to 17.8 MPa, while the corresponding values at 7 and 28 days decreased from 29.1 to 27.5 MPa and from 42.3 to 39.0 MPa, respectively. These results suggest that construction delay alone can adversely affect the hardened performance of mortar even without the addition of extra water.

The reduction in compressive strength became considerably more pronounced after re-tempering. For mixtures containing 2% additional water, the compressive strengths at 3 days were 17.2, 15.5, and 13.2 MPa after waiting times of 15, 30, and 45 min, respectively. The corresponding strengths at 7 days were 26.8, 24.5, and 21.0 MPa, while those at 28 days decreased to 37.5, 34.0, and 29.5 MPa. When the additional water content was increased to 4%, strength reductions became even greater, with 28-day compressive strengths decreasing to 35.2, 31.2, and 26.0 MPa for waiting times of 15, 30, and 45 min, respectively. These observations indicate that the adverse effect of re-tempering appears at early ages and persists throughout the later curing period.

The opposite trends observed for flow recovery and compressive strength became increasingly evident with increasing waiting time. For example, under the 2% re-tempering condition, the flow increased by 17 mm (169–186 mm) after a waiting time of 15 min, whereas the corresponding 28-day compressive strength decreased by only 2.5 MPa (40.0–37.5 MPa). At a waiting time of 30 min, however, the flow recovery decreased to 12 mm, while the strength reduction increased to 5.5 MPa. After 45 min, the flow recovered by only 9 mm, whereas the compressive strength decreased by as much as 9.5 MPa. A similar tendency was observed for the mixtures containing 4% additional water, where larger improvements in workability were accompanied by greater reductions in compressive strength.

These findings demonstrate that the dominant effect of re-tempering evolves with elapsed time. Although the increase in the nominal water-to-cement ratio was relatively small (from 0.50 to approximately 0.51–0.52), the strength reduction cannot be explained solely by this change. Rather, the results suggest that the combined effects of delayed water addition, progressive particle flocculation, and disturbance of the developing hydration structure contributed to the observed deterioration in hardened performance [7,9,11,25,26]. At short waiting times, the beneficial effect of workability recovery is relatively significant. As the waiting time increases, however, the improvement in workability gradually diminishes, whereas the deterioration of hardened properties becomes increasingly severe. Accordingly, the dominant effect of re-tempering shifts progressively from workability recovery toward deterioration of hardened properties, representing a time-dependent transition.

### 3.3. Time-Dependent Transition of Re-Tempering Behavior

**Figure 3:**
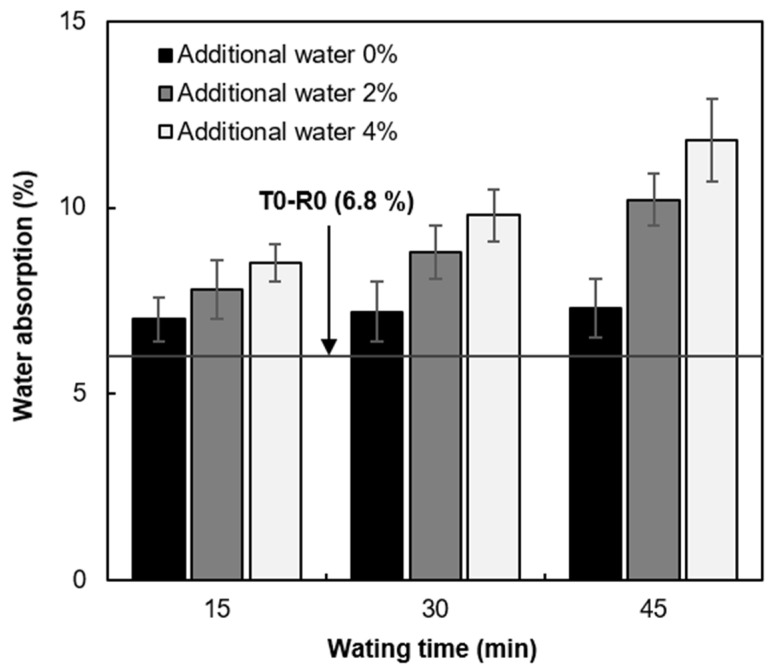
Water absorption of mortar after 28 days as a function of waiting time and additional water content. The solid horizontal line represents the water absorption of the reference specimen (T0-R0). Error bars represent standard deviations (*n* = 3).

Figure 3 presents the water absorption of mortar after 28 days as a function of waiting time and additional water content. For the control mixtures (R0), water absorption increased only slightly from 6.8% to 7.3% as the waiting time increased from 0 to 45 min. This observation indicates that construction delay alone has a limited influence on the moisture transport characteristics of hardened mortar.

By contrast, the re-tempered mixtures exhibited substantially higher water absorption. With 2% additional water, the absorption increased to 7.8%, 8.8%, and 10.2% after waiting times of 15, 30, and 45 min, respectively. When the additional water content was increased to 4%, the absorption further increased to 8.5%, 9.8%, and 11.8%.

When interpreted together with the compressive strength results, a consistent trend becomes apparent. As the additional water content increased, compressive strength decreased, whereas water absorption increased. For example, at a waiting time of 45 min, increasing the additional water content from 0% to 4% reduced the 28-day compressive strength from 39.0 MPa to 26.0 MPa, corresponding to a reduction of approximately 33%, while the water absorption increased from 7.3% to 11.8%, representing an increase of approximately 62%.

These results suggest that re-tempering reduces the compactness of the hardened cementitious matrix, thereby simultaneously decreasing compressive strength and increasing water absorption. This trend is generally attributed to the increase in capillary porosity associated with a higher effective water-to-cement ratio, which facilitates water transport through the hardened mortar [4,16,17,18,27]. However, since no microstructural characterization was performed in the present study, the underlying mechanisms responsible for these changes require further investigation.

Taken together, the results presented in Figure 1, Figure 2 and Figure 3 demonstrate that re-tempering simultaneously affects both fresh and hardened properties. Nevertheless, evaluation of each property individually is insufficient for quantitatively assessing the balance between workability recovery and deterioration of hardened performance. Therefore, a comparative indicator is introduced in the following section to evaluate this balance quantitatively.

### 3.4. Quantitative Assessment of Time-Dependent Transition

Re-tempering inherently involves a trade-off between the recovery of workability and the deterioration of hardened performance. To quantitatively evaluate the relative balance between these competing effects, a dimensionless Workability–Strength Trade-off Ratio (WSTR) was defined, as expressed in Equation (2):

(2) $$WSTR=\;\frac{∆Flow/{Flow}_{R0}}{∆f_{c,28}/f_{c,R0}}$$

where *ΔFlow* denotes the increase in flow produced by re-tempering under the same waiting time, and *Flow_R_*_0_ is the flow of the corresponding control mixture without re-tempering. Likewise, *Δf_c_*_,28_ represents the reduction in 28-day compressive strength caused by re-tempering, and *f_c_*_,_*_R_*_0_ is the corresponding strength of the control mixture. WSTR is defined only for re-tempered mixtures and is calculated relative to the corresponding non-re-tempered mixture at the same waiting time. The control mixtures themselves are not assigned WSTR values because both normalized changes are zero. Furthermore, WSTR is not defined when the reduction in 28-day compressive strength is zero. In all re-tempered conditions investigated in the present study, both *ΔFlow* and *Δf_c_*_,28_ were positive, resulting in finite WSTR values. Consequently, WSTR represents the ratio between normalized workability recovery and normalized 28-day compressive strength loss.

Unlike an absolute performance index, WSTR is intended as a dimensionless comparative indicator for evaluating the relative efficiency of different re-tempering conditions within the same experimental framework.

As summarized in Table 2, WSTR consistently decreased with increasing waiting time regardless of the amount of additional water. For mixtures containing 2% additional water, WSTR decreased from 1.62 at 15 min to 0.55 and 0.25 at waiting times of 30 and 45 min, respectively. Similar reductions were observed for mixtures containing 4% additional water, where WSTR decreased from 1.43 to 0.76 and 0.51.

These results indicate that, at short waiting times, the relative benefit of workability recovery is greater than the relative penalty associated with strength loss. Although the normalized workability recovery remained nearly constant under the 4% re-tempering condition, the normalized strength loss increased consistently with waiting time. Consequently, the continuous decrease in WSTR was governed primarily by the progressive increase in normalized strength loss rather than by a continuous reduction in workability recovery.

In particular, a noticeable reduction was observed around the investigated waiting time of 30 min, suggesting that the engineering benefit of re-tempering tends to decrease as the waiting time increases. Because only three delayed waiting times were investigated in the present study, this observation should be interpreted as a tendency within the investigated experimental range rather than as evidence of a definitive transition threshold. It should also be noted that all investigated re-tempering times (15–45 min) were within the pre-setting stage of the mortar under the present experimental conditions. Therefore, the observed time-dependent transition should be interpreted as an early hydration phenomenon rather than as a transition directly associated with the initial or final setting of cement.

It should be emphasized that WSTR is not proposed as a universal material property or performance index. Instead, it serves as a comparative indicator for evaluating the relative balance between workability recovery and strength deterioration under identical mixture proportions and testing conditions. Although its applicability to other mortar systems requires further validation, WSTR provides a convenient quantitative framework for integrating the effects of fresh and hardened properties and supports the interpretation of re-tempering as a time-dependent transition rather than an isolated change in individual material properties.

## 4. Conclusions

This study investigated the effects of waiting time and re-tempering water content on the fresh and hardened properties of cement mortar under controlled laboratory conditions. Based on the experimental results, the re-tempering behavior was interpreted from the perspective of a time-dependent transition. The main findings are summarized as follows:

(1) The flow of mortar continuously decreased with increasing waiting time, even without re-tempering. The addition of extra water effectively restored the lost workability; however, the recovery became progressively less effective as the waiting time increased.

(2) Re-tempering reduced the compressive strength at all curing ages, and the magnitude of strength loss became more pronounced with increasing waiting time and additional water content. Furthermore, water absorption after 28 days increased consistently under the same conditions, confirming the trade-off between workability recovery and deterioration of hardened properties.

(3) A dimensionless Workability–Strength Trade-off Ratio (WSTR) was introduced as a comparative indicator to evaluate the relative balance between normalized workability recovery and normalized strength loss. WSTR decreased consistently with increasing waiting time for all re-tempering conditions, with a noticeable reduction around the investigated waiting time of 30 min. These results suggest that the relative engineering benefit of re-tempering gradually diminishes as the waiting time increases within the investigated experimental range.

(4) The combined evaluation of flow, compressive strength, water absorption, and WSTR demonstrates that the dominant effect of re-tempering gradually shifts from workability recovery to deterioration of hardened properties as the waiting time increases. These findings support the interpretation of re-tempering as a time-dependent transition, rather than as an isolated change in individual material properties.

Unlike previous studies that evaluated re-tempering primarily through individual properties such as workability or compressive strength, the present study integrated fresh and hardened performance using a comparative indicator to quantitatively assess their relative balance. Although the proposed WSTR is not intended to represent a universal performance index, it provides a practical framework for comparing the efficiency of different re-tempering conditions within the same experimental system.

This study is limited to a single mortar mixture and controlled laboratory conditions, and no microstructural characterization was performed. Future studies involving a wider range of waiting times, different binder systems, environmental conditions, statistical analyses, and microstructural characterization are required to validate the proposed interpretation and to establish a more generalized framework for evaluating re-tempering behavior. Additional experimental data will also be necessary to determine whether a more definitive transition point or criterion exists. In addition, more comprehensive statistical analyses will be essential to further strengthen the reliability and generalizability of the proposed interpretation.

## Figures and Tables

**Table 1:** Mix design and experimental variables.

Mix ID	C:S (by Mass)	W/C Ratio	Waiting Time (min)	Additional Water (% of Initial Mixing Water)
T0-R0	1:3	0.5	0	0
T15-R0			15	0
T30-R0			30	0
T45-R0			45	0
T15-R2			15	2
T30-R2			30	2
T45-R2			45	2
T15-R4			15	4
T30-R4			30	4
T45-R4			45	4

Note: C = cement, S = sand, and W = water.

**Table 2:** Normalized flow recovery, normalized 28-day strength loss, and WSTR of re-tempered mortar.

Waiting Time (min)	Additional Water (%)	Normalized Flow Recovery	Normalized Strength Loss	WSTR
15	2	0.101	0.063	1.62
30	2	0.077	0.139	0.55
45	2	0.061	0.244	0.25
15	4	0.172	0.120	1.43
30	4	0.160	0.210	0.76
45	4	0.169	0.333	0.51

## Data Availability

The original contributions presented in this study are included in the article. Further inquiries can be directed to the corresponding author.
